# Optimal Workloop Energetics of Muscle-Actuated Systems: An Impedance Matching View

**DOI:** 10.1371/journal.pcbi.1000795

**Published:** 2010-06-03

**Authors:** Waleed A. Farahat, Hugh M. Herr

**Affiliations:** 1 Department of Mechanical Engineering, Massachusetts Institute of Technology, Cambridge, Massachusetts, United States of America; 2 The Media Laboratory and MIT - Harvard Division of Health Sciences and Technology, Massachusetts Institute of Technology, Cambridge, Massachusetts, United States of America; Northwestern University, United States of America

## Abstract

Integrative approaches to studying the coupled dynamics of skeletal muscles with their loads while under neural control have focused largely on questions pertaining to the postural and dynamical stability of animals and humans. Prior studies have focused on how the central nervous system actively modulates muscle mechanical impedance to generate and stabilize motion and posture. However, the question of whether muscle impedance properties can be neurally modulated to create favorable mechanical energetics, particularly in the context of periodic tasks, remains open. Through muscle stiffness tuning, we hypothesize that a pair of antagonist muscles acting against a common load may produce significantly more power synergistically than individually when impedance matching conditions are met between muscle and load. Since neurally modulated muscle stiffness contributes to the coupled muscle-load stiffness, we further anticipate that power-optimal oscillation frequencies will occur at frequencies greater than the natural frequency of the load. These hypotheses were evaluated computationally by applying optimal control methods to a bilinear muscle model, and also evaluated through *in vitro* measurements on frog *Plantaris longus* muscles acting individually and in pairs upon a mass-spring-damper load. We find a 7-fold increase in mechanical power when antagonist muscles act synergistically compared to individually at a frequency higher than the load natural frequency. These observed behaviors are interpreted in the context of resonance tuning and the engineering notion of impedance matching. These findings suggest that the central nervous system can adopt strategies to harness inherent muscle impedance in relation to external loads to attain favorable mechanical energetics.

## Author Summary

Movement in organisms is a result of the interplay between biomechanics, neural control, and the influence of external environmental loads. Understanding the interaction between these factors is important not only for scientific reasons but also for engineering robotic systems and prostheses that strive to match biological performance. Muscle mechanical impedance is key in defining the mechanical interaction between muscles and their loads. It is well known that neural activation modulates muscle impedance, particularly stiffness, and that such modulation can be used advantageously to stabilize the posture and motion in organisms. Here, we show computationally and experimentally that stiffness modulation can also be used to enhance the capability of muscle to generate mechanical power, which is key in determining how fast animals can run, fly, swim, or jump. When muscles are activated optimally in relation to their external loads, they can create resonance conditions at optimal frequencies that significantly enhance their mechanical energetics by up to 7-fold. These findings can be interpreted in the context of the engineering notions of impedance matching and resonance tuning, which are commonly used as guiding principles in the design of diverse power optimal systems, such as communication circuits and robotic systems.

## Introduction

The capability of skeletal muscles to deliver mechanical power is key in determining the neuromechanical performance envelope of organisms. How fast and how far animals run, fly, swim, or jump is clearly limited by the mechanical power delivered by the muscle-tendon units to skeletal and environmental loads. Therefore, estimating the mechanical energetics of muscles (henceforth simply called energetics) has been of interest in diverse fields such as organismal biomechanics, biomimetic robotics and prosthetics [1]–[3].

Many factors influence the neuromechanical performance of organisms, including i) the dynamics and mechanical properties of muscle actuators, ii) skeletal mechanics, iii) neural control and iv) influence of loads external to the organism. Integrative approaches have been proposed to capture the interaction of all, or subsets of these factors. For example, the connection between muscle impedance (particularly stiffness) and neural control has been studied in depth with respect to postural and dynamic stability [4], [5], locomotory functions [6]–[9], manipulation [10], [11], and other biomechanical tasks [12]. In this work, we adhere to the definition of muscle mechanical impedance as the “static and dynamic relation between muscle force and imposed stretch” [4]. Muscle impedance encompasses muscle stiffness, which is the static relation between muscle force stretch only.

In the context of muscle energetics, most investigations focused on experimentally measuring the power output of individual muscles at a range of frequencies, phases and electrical stimulation parameters, and finding maximal power generating capability of muscles under prescribed motion trajectories. However, the role of muscle-load interaction on output energetics has not been formalized. The central premise of this work is that the mechanical energetics of a muscle-actuated system cannot be determined in a meaningful manner without considering the coupling of muscle properties, load dynamics and neural activation. By considering this coupling explicitly, we arrive at phenomena that cannot be captured using standard workloop testing methodologies, including the opportunity to harness muscle-load interaction in an energetically advantageous manner.

Muscle energetics have been characterized under dynamic conditions, both *in vitro* [13] and *in vivo* [9], [14], [15]. *In vitro* measurements relied almost invariably on the workloop technique [16]. In this approach, isolated muscles are subjected to predetermined periodic length variations in time (typically sinusoidal, but not always [17]) by means of an external motion source. At a given phase of the imposed oscillation, an electrical stimulus is delivered synchronously, resulting in periodic muscle contractions. A plot of muscle contractile force versus displacement results in a cyclic workloop, with the integrated area within the loop being a measure of the net muscle work done. These and similar measurements have been reproduced in the muscle physiology literature for various muscle groups within various organisms [18]–[21], and connections between the muscle function and its mechanical energetics have been made [22]–[24]. While such measurements provide useful energetic connections with muscle function, the experimental conditions do not capture representative *in vivo* conditions because motion profiles are imposed on single isolated muscles with no muscle-load interactions [25], and without incorporating the effects of antagonist activity. *In vivo* measurements, on the other hand, capture all of the above effects in principle, but lack the experimental flexibility of varying load conditions in an unambiguous manner.

Capturing the effect of muscle-load interaction on muscle energetics is critical. This interaction can be captured by considering the impedance of the muscles in relation to the impedance of the load. When a group of muscles acts on a common load, as exemplified by an antagonist pair acting on a common load, each muscle forms part of the load borne by the other muscles in its group. Because muscle impedance is activation dependent, neural control can be used to modulate the effective load observed by each muscle by modulating the impedance of the opposing muscles, thereby offering the opportunity to create favorable impedance conditions that maximize power transfer to the external environmental load. This is akin to the notion of impedance matching in engineering systems, where the driving source and the load are “matched” to provide optimal power transfer. In the context of neuromuscular control, impedance matching can enable groups of muscles to work synergistically to provide significantly higher energetics than the sum of individual muscles.

**Figure 1:**
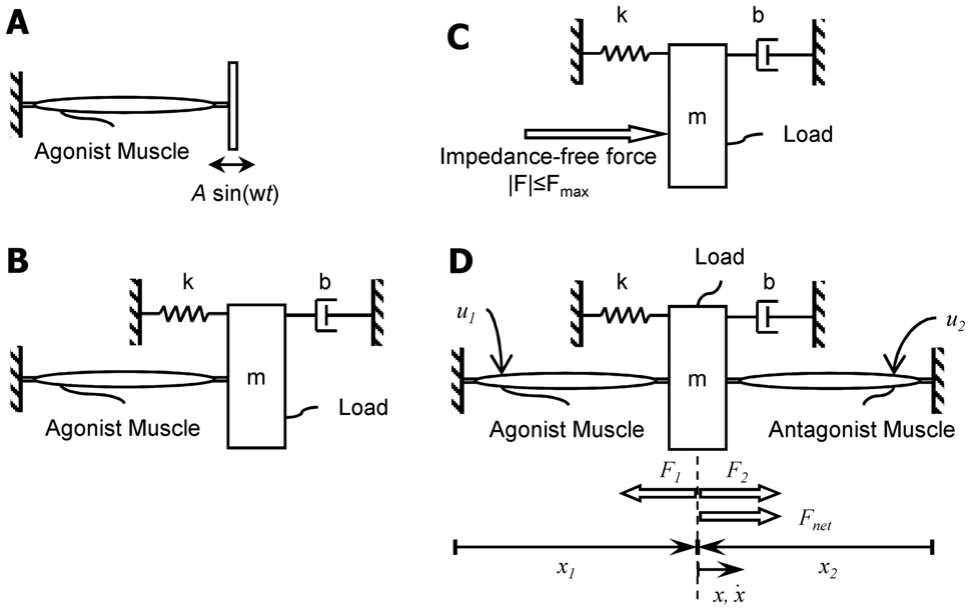
Problem cases illustrating the role of muscle-load interaction. (A) Standard setting of a workloop experiment where a muscle acts against a non-admitting motion source (sinusoidal in this case). (B) A single muscle acting against an admitting load (a mass-spring-damper system in this example, with mass 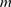, stiffness 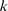 and damping constant 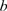). (C) An idealized, impedance-free force source acting on the same load. The force source is limited in absolute magnitude by 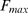. (D) Antagonist muscles acting against a common admitting load. In this setup, muscles communicate with each other mechanically through the common load. The impedance of one muscle forms part of the load of the other. Note that in (A) the motion is imposed on the muscle irrespective of its contractile force, while in (B), (C) and (D) cyclic motions result from applied muscle or actuator forces. The coordinates and definitions of the variables used for system modeling are shown in panel (D). The contractile forces are 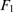 and 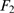 for the agonist and antagonist muscles respectively, whereas the net force is 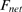. The lengths of the muscles is 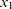 and 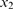, and the variables 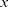 and 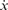 are in the load reference frame. The electrical stimulus delivered to the muscles comprises the system input, and is indicated by 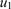 and 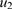.

Consequently, in this investigation we studied the influence of muscle-load interaction on muscle workloop energetics both computationally and experimentally. We set up a model problem consisting of a mass-spring-damper system actuated by either a single muscle (Figure 1B), or a pair of symmetric, antagonist muscles (Figure 1D). The input to the system (either neural control or electrical stimulation) can modulate the net force exerted by the two muscles as well as the net impedance. In the context of this problem, we investigated two hypothesis. Hypothesis 1 states that the power optimal oscillation frequency of a muscle actuated system is greater than the resonance frequency of the load. This is in direct contrast to an impedance-free actuator (such as an ideal electric motor) where the optimal oscillation frequency occurs exactly at the resonance of the load. Hypothesis 2 states that a pair of antagonist muscles can work together to produce more power synergistically than individually by margins that cannot be predicted without explicit incorporation of muscle impedance. We tested these hypotheses both computationally and experimentally. Our computational approach relied on optimal control solutions to the workloop maximization problem, which was based on a mathematical model of the problem. The experimental approach relied on *in vitro* measurements of workloop energetics of electrically-stimulated, frog muscle acting against emulated mass-spring-damper loads.

## Materials and Methods

### System Model

To investigate the role of muscle-load interaction and muscle impedance on output energetics, a mathematical model of the problem was developed. This model formed the basis for the ensuing optimization of workloop energetics. We modeled the case of Figure 1D. Note that the case of Figure 1B is a special case of the problem considered with the coefficients of the antagonist muscle set to zero. The key ingredient is a muscle model that captures activation and impedance characteristics of the muscle.

#### Excitation-contraction dynamics

We assumed the excitation-contraction dynamics had temporal responses that were of the same time scale as that of the oscillatory periods, and consequently cannot be neglected. These dynamics capture the rise and fall of muscle force in time as inputs are applied. They were captured by second order processes with real poles that model calcium diffusion dynamics. We assumed the following model:
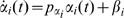
(1)

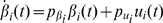
(2)where 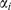 is the activation state of muscle 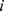, 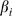 is an intermediate state of Ca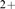 diffusion and re-uptake dynamics and 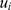 is the electrical stimulus input to muscle 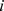. The parameters 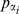, 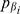 and 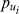 were estimated based on temporal twitch profiles (as detailed in the supporting material Text S1). The parameters used resulted in simulated twitch rise and fall time of 125 msec, and a gain of unity.

#### Bilinear muscle force model

We assumed that the contractile force exerted by muscle 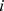, 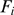, can be approximated by the function
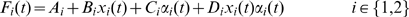
(3)


In this model, muscle force 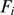 is bilinear in length 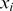 and activation 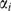. Consequently, the muscle stiffness 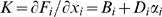 is linear in activation. The parameters 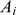, 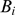, 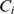 and 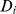 were identified based on experimental characterizations that are described in the supporting material Text S1 and illustrated in Figure S1. Similar bilinear models have been used to describe muscle force production in relation to EMG signals in the upper arm [4], [26], and also with respect to steady-state force production in electrically stimulated cat soleus muscles [27]. In other work [28], we found that for cyclic, bursting contractions, the bilinear model captures 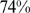 of the variance in muscle force production over independent validation sets.

#### Net muscle force exerted on load

Since the contractile force of each muscle was described with respect to its local coordinates, we used the following transformation:
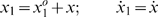


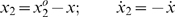
where 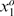 and 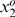 are the nominal lengths of the muscles. Therefore, the net muscle force is:
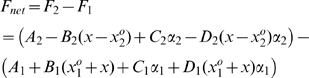
(4)


#### Load dynamics

The net muscle force excites the mass-spring-damper system and the resulting response is characterized by:
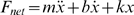
(5)


#### Interconnected system state equations

From Equations (1), (2), (3), (4), and (5) the dynamics of the interconnected system are written as:
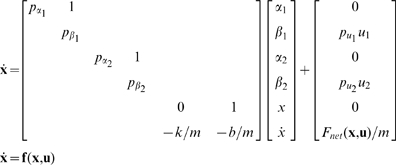
(6)where the state vector is 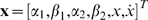 and the control input vector is 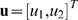. The nonlinearity of the system is captured by the bilinear nature of 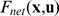.

### Optimization of Muscle Workloop Energetics

The model of Equation (6) was treated as the basis for our analysis. Since our objective is to analyze optimal muscle workloop energetics, we maximize the average power transfer from the muscles to the load integrated over one periodic cycle. The instantaneous power delivered to the load is given by 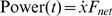. The cyclic work done by the muscles on the load is the integral of the power over one complete cycle. Therefore the control inputs, 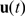, that characterize power-optimal oscillations are given by the solution of the following optimization problem:
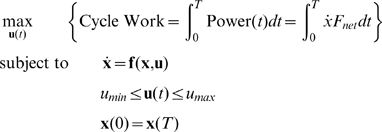
(7)where 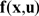 is defined in Equation (6) and 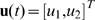 is the control input vector. In this formulation, we assumed that the terminal time 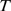 was given and defined by the objective task. Therefore, to optimize power at oscillations of frequency 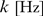 [Hz], we set the solution time horizon 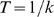 [sec].

To derive necessary conditions for the optimal solution of Problem (7), we applied the Pontryagin Minimum Principle [29]. We followed the following procedure:

1. Augment the cost function with multipliers for each of the constraints.
2. Define the Lagrangian and Hamiltonian scalar functions.
3. Write the equations governing the dynamics of the optimal multipliers.
4. Define the necessary conditions for optimal control.
5. Solve the resulting 2-point boundary value problem for the optimal state trajectory and the associated multipliers.

Details of this derivation, and the numerical methods employed therein are described as follows. The integrand of the Lagrangian cost function 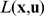 is given by
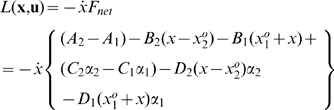
We augment the dynamical constraints to the cost function, and define the Hamiltonian scalar function
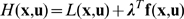
From the Pontryagin principle [29], the evolution of the optimal co-state variables at the optimal solutions are governed by:
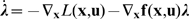



The optimal control 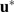 is given by
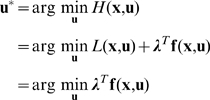
where the last equality follows since 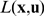 is not a function of 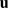 in this particular context. Substituting in Equation (6), we get
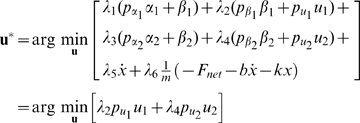
which implies
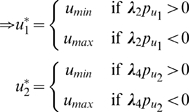
where 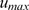 and 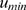 are upper and lower bounds, respectively, on the control inputs. Depending on the signs of the switching functions 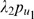 and 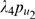, the control 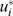 assumes either the values 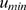 or 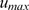. This is a *bang-bang control* solution, and is an expected outcome in such power-optimal (or maximum acceleration) problems [30]. Mathematically, such solutions appear when the Hamiltonian 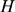 is a linear function in the control 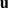, as is the case in this problem. In the absence of limits on the control, the optimization problem would be unbounded, implying that the muscles that can generate unbounded forces will add infinite power to the load. Therefore, for the optimization problem to be mathematically well-posed, upper and lower bounds on the control inputs 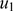 and 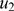 are necessary.

In summary, the first order necessary conditions for power-optimal solutions are given by:
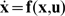
(8)

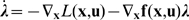
(9)

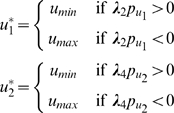
(10)with cyclic boundary conditions:
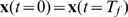
(11)

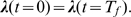
(12)


Equations (8) and (9) define a two-point boundary value problem (2-point BVP) that is subject to the cyclic boundary conditions (11) and (12) and control constraints (10). This 2-point BVP was solved to give the optimal state trajectory (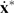), the optimal control inputs 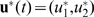, and the multipliers (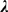) associated with the power optimal solution. Methods for solving this problem numerically are detailed in the supporting material Text S1.

### Experimental Methods

#### Ethics statement

All animals were handled in strict accordance with good animal practice as defined by the relevant national and/or local animal welfare bodies, and all animal work was approved by the MIT Committee on Animal Care (protocol number 0705-051-08).

#### Experimental framework

Experimental investigations played a key verification role in this work in measuring muscle workloop energetics under the conditions illustrated in Figure 1B and 1D, as well as in generating data sets to identify mathematical muscle models (Equations (1), (2) and (3)) necessary for ensuing optimizations [28]. A full description of the experimental platform and techniques can be found in [25]. For the benefit of the reader, we provide a brief description here.

Explanted muscle experiments were conducted on *Plantaris longus* muscles harvested from adult male *Rana pipiens* (leopard frogs). These muscles were chosen primarily for ease of dissection of two contralateral muscles from the same frog, and because their extremal points provide natural mechanical interfaces to the experimental apparatus (specifically the Achilles tendon and the knee joint). Experiments were performed on single muscle configurations as well as configurations of muscle pairs acting antagonistically as shown in Figures 1B and 1D. These arrangements were achieved by connecting the muscles to load-emulating servo-systems. The servo systems measured the muscle contractile force, and imposed a position trajectory in accordance with the dynamics of the modeled load in real-time, thereby effectively connecting the muscle to mechanical boundary conditions mimicked. In the case of Figure 1D, the interaction of two antagonist muscles acting on a common load was achieved by linking two separate servo-systems in software, i.e. the net measured force (agonist muscle contractile force minus antagonist muscle contractile force) drove the virtual load. Thus, when one muscle extended, the other contracted simultaneously and commensurately in accordance with the equations of motion for the load (mass-spring-damper in this case). This setup allowed for direct interaction between the muscle under evaluation, the load, and the opposing muscle. It also enabled clear experimental separation of load and muscle dynamics where different load parameters could be easily programmed in software. Additionally, this platform enabled direct electrical stimulation of the muscle through the sciatic nerve which remained unsevered during dissection. The muscles were placed in oxygenated, circulating amphibians Ringer's solution to maintain viability during the course of experiments. All experiments were conducted at room temperature (approximately 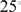
C), though the temperature was not explicitly controlled. In the cases of antagonist muscles (Figure 1D), experiments were performed on contralateral muscle pairs harvested from the same frog, thereby maximizing similarity between the two muscles.

Bipolar electrical stimulation was delivered to the muscles via hook electrodes that were in contact with the sciatic nerve. Since the efficacy of electrical stimulation depends on the contact resistance of the nerve and the electrode (which varies for each experimental session), the voltage of the simulation trains was gradually increased at the beginning of each muscle until full recruitment was observed (as determined by saturation in the amplitudes of isometric twitch force profiles). Stimulation was provided in waveforms repeating at the desired oscillation frequency of the mechanical system. During the active segments of the waveform, the muscles were stimulated with a pulse train at 200 Hz to ensure full recruitment, and a pulsewidth of 100 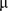
s. The duration of the active segments of the pulse train and the oscillation frequency (waveform frequency) were determined based on the solutions of the optimal control problem.

#### Experimental conditions

To evaluate the hypotheses, we measured workloop energetics of single muscles and muscle pairs acting against emulated mass-spring-damper system. To evaluate Hypothesis 1, we compared the power output of a single muscle acting on the mass-spring-damper system at three frequencies: i) 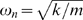, the natural frequency of the load, ii) 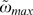, an estimate of 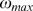, the frequency that attains maximal power as predicted by the model, where 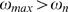, and iii) 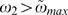, a third frequency that is distinctly higher. We conducted measurements using the parameters {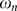 = 2 Hz, 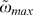 = 2.5 Hz, 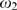 = 3 Hz} for 3 test muscles and the using the parameters {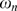 = 4 Hz, 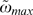 = 5 Hz, 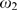 = 6 Hz} for 4 test muscles. Workloop measurements were conducted in sets measuring the power output at the three different frequencies, i.e. 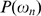, 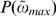, and 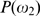. To factor out any potential confounding effects due to muscle fatigue, the order of the measurements in each set was randomized, and each measurement was normalized by 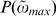 of each corresponding set. The normalized power values are denoted by 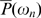, 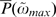 and 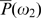. For each muscle, measurements were repeated 5–7 times, with each measurement consisting of the average of 7 oscillatory cycles. The power estimate from the first cycle discarded because it is an atypical oscillation that does not follow the steady-state trajectory since the system starts from rest.

To evaluate Hypothesis 2, we compared the sum of the powers generated by each muscle individually to the power generated by two muscles working together on the same load. Here, measurement sets consisted of 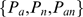, where 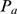 is the power generated by the agonist muscle only, 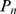 is the power generated by the antagonist muscle only, and 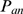 is the power generated by both muscles working in concert. Similar to the treatment of the data pertaining to Hypothesis 1, measurements were also randomized in their order to factor out the effects of fatigue. The synergistic comparison is captured by the ratio
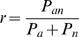
which is computed for each data set. For each muscle pair, measurements were repeated 6 times, with each measurement consisting of the average of 7 oscillatory cycles (with the the the first cycle discarded as well). We conducted measurements on a load having 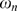 = 2 Hz, with oscillation frequencies set to 3 Hz (3 muscle pairs) and 4 Hz (4 muscle pairs).

In both sets of experiments, the setting of the natural frequency of the load (2–4 Hz) was comparable to frog jumping frequencies (observed at 2 Hz [31]) and frequencies of high muscle power output using the standard workloop technique (observed at 4 Hz [18]). Load stiffness was chosen to be comparable with muscle stiffness (750 N/m to 1500 N/m), and mass and damping ratios (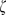) were chosen to limit the amplitude of muscle strain to within experimentally viable ranges.

## Results

### Optimization Results

**Figure 2:**
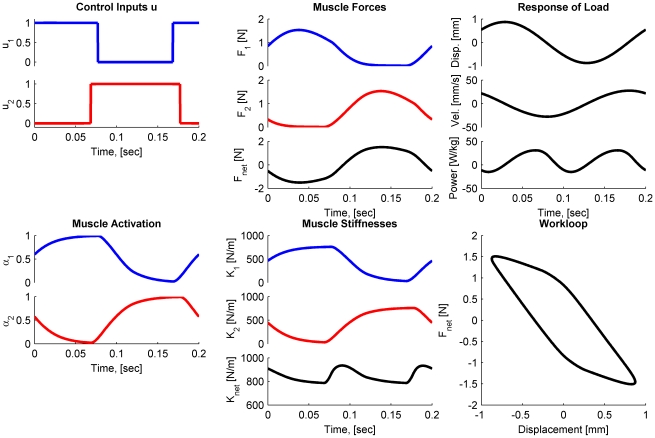
Solution of the optimal control problem. This is an example solution for a time horizon of 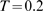 seconds, corresponding to an oscillation frequency of 5 Hz. Plots show the bang-bang control inputs [dimensionless] and corresponding activation [dimensionless], muscle forces [N], time-varying stiffnesses [N/m], the motion of the mass-spring-damper system (displacement, velocity, and the net power imparted per kg of muscle), and the resulting workloop. In the plots, blue traces pertain to the agonist muscle, the red traces to the antagonist muscle, and black traces refer to the net effects of both muscles and the load. Note that this particular solution exhibits co-activation as evidenced by the degree of overlap in the activation signals, and also in the control signals. This co-activation was required to stiffen the overall system to accommodate the relatively high-frequency of excitation required.

The optimal control problem (Problem (7)) was solved for various values of the time horizon 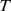 that characterized the oscillation frequencies of interest. An example solution is shown in Figure 2 for an oscillation frequency (5 Hz) that is greater than the load resonance frequency (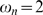 Hz).

**Figure 3:**
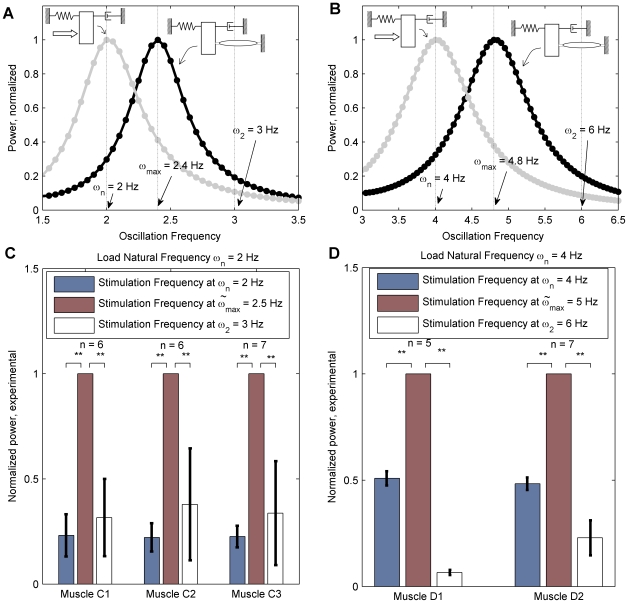
The optimal stimulation frequency (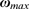) for a mass-spring-damper system actuated by a muscle is greater than the natural frequency of the load (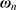). (A & B) Results of dynamic optimizations. Each point in the plots represents a solution similar to that of Figure 2. For an impedance-free actuator (gray), the optimal frequency coincides with the load natural frequency, whereas for the bilinear muscle model incorporating activation dependent stiffness (black) the optimal frequency is greater. Results are shown for simulations with 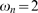 Hz, 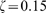 and 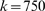 N/m (A), and 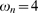 Hz, 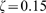 and 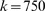 N/m (B). (C & D) Experimental measurements of power ratios shown for each measurement set. Workloop power measurements in each set are normalized by 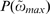. The error bars at 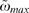 are therefore equal to zero by definition. Both figures show that 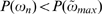 and 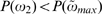. The asterisks indicated the 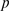 value, with (**) for 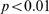 and (*) for 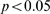. (C) Measurements taken across 3 muscle for load natural frequency 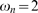 Hz, 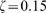 and 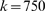 N/m. (D) Measurements taken across 2 muscles for load natural frequency 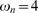 Hz, 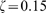 and 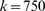 N/m.

To investigate Hypothesis 1 computationally, successive optimizations similar to those of Figure 2 were conducted as the oscillation frequency was swept across the range of interest, and comparisons between optimal power generated by the bilinear muscle model and the optimal power generated by an impedance free actuator were drawn. As shown in Figure 3A, in the case of the system with 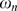 = 2 Hz, the peak power was generated at 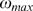 = 2.4 Hz. In Figure 3B, in the case with 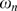 = 4 Hz, the peak power was at 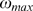 = 4.8 Hz. This result is in direct contrast to the case when the load is driven by impedance-free actuators, where the optimal driving frequency is exactly equal to the resonance frequency of the load. The increase in optimal stimulation frequency is attributed to the contribution of active muscle stiffness to the net stiffness of the system (shown in the stiffness sub-plots of Figure 2), and thereby tuning the resonance of the combined muscle-load system.

**Figure 4:**
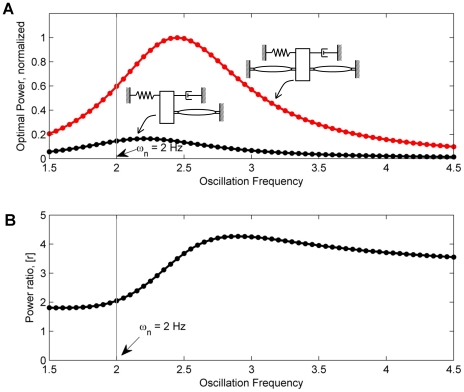
Summary of optimal solutions as a function of oscillation frequency. Each point the plots represents a solution similar to that of Figure 2. (A) Maximal power output produced by antagonist muscles (red) and by an individual muscle (black). (B) Energetic synergies in workloop measurements can be explored by comparing the ratios 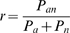, which at certain frequencies is substantially higher than 2. 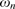 is the resonance frequency of the mass-spring-damper unloaded by the muscles. Results shown for 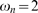 Hz, 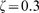 and 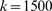 N/m.

To investigate Hypothesis 2 computationally, we compared the power output of the optimal solutions of the single-muscle case against the optimal solutions of the case of a muscle pair in Figure 4 across the frequency range of interest. The computed power-optimal responses show that synergistic activation of antagonist muscles may produce more cyclic work than individual muscle activation by a factor of more than two (Figure 4B). This is captured by the synergistic ratio 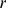, and is in direct contrast to constant impedance actuators where the ratio is exactly two. This model prediction implies that the energetics of individual muscles (obtained by zero-admittance workloop tests) cannot simply be summed to draw conclusions regarding the workloop energetics of the entire system.

### Experimental Workloop Energetics

Figures 3C and 3D show the results of experimental workloops with single muscles acting on mass-spring-damper loads. To test Hypothesis 1 experimentally, that the peak normalized power output was indeed at 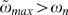, measurements were conducted on two load cases with different natural frequencies (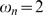
Hz and 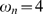
Hz). For both loads, we found that the normalized power measures 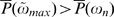 and 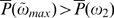, with (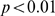 for all measurements). We attribute this increase in the optimal oscillation frequency over 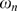 to the stiffness contribution of the muscles. This increase in optimal frequency over 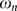 cannot be achieved via an impedance free force source, and can therefore be directly attributed to the increase in muscle stiffness due to the activation profile over the course of a full cycle.

**Figure 5:**
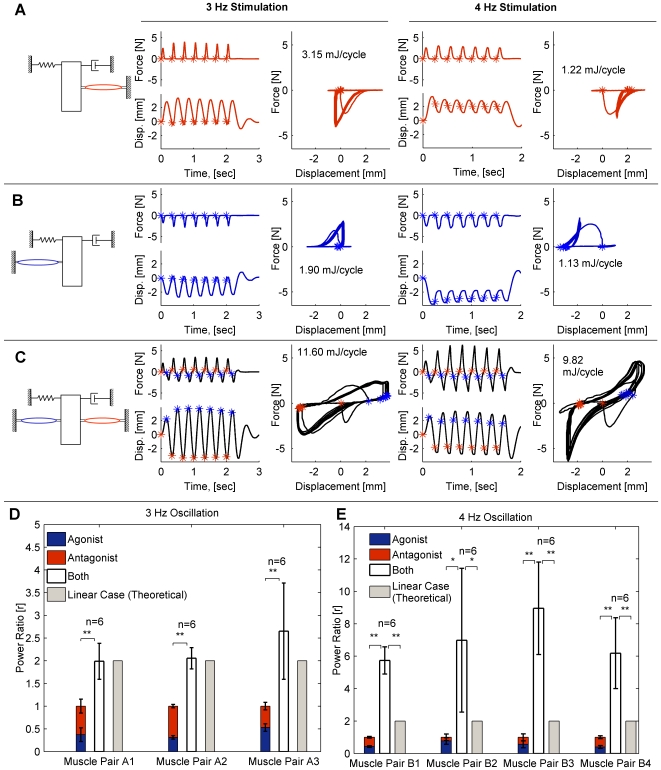
Experimental arrangements and workloops of agonist and antagonist muscles acting on second-order loads. At certain frequency ranges, the optimal workloop energetics of a pair of antagonist muscles acting in concert is more than the optimal workloop energetics of the muscles acting individually by a factor of 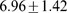 (representing mean and standard deviation of the averages of 4 muscle pairs). (A) Workloops of agonist muscle acting individually. (B) Workloops of antagonist muscle acting individually. (C) Workloops of muscle pair stimulated out of phase, producing more work on the same load. Asterisks indicate stimulation points (red is for agonist, blue is for antagonist). The first workloop is atypical as the system converges to a steady-state response and is discarded from energetic computations. All workloops have a counter clock-wise direction, indicating positive muscle work, which equals the energy dissipated in the damper. For all cases the natural frequency of the load was 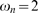 Hz, 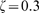 and 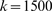 N/m. (D) Compiled results for data points similar to A, B and C, with operating frequency = 3 Hz. Data shown across 3 muscle pairs. The workloop energetics of the two muscles working together is not statistically significant from a value of 2 predicted in the theoretical case of linear, impedance-free actuators. (E) Compiled results for data points similar to A, B and C, with operating frequency = 4 Hz. Data shown across 4 muscle pairs. The workloop energetics of the two muscles working together significantly greater than 2. The asterisks indicated the 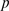 value, with (**) for 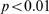 and (*) for 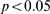.

Figure 5 shows the power output measurements of a pair of antagonist muscles acting synergistically compared to their power output acting individually. When the oscillation frequency was set to 3 Hz, the value of the energetic ratio 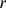 was not statistically different from 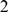. However, when the oscillation frequency was set to 4 Hz, we found 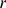 to be 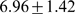. The ratio 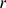 was significantly greater than 2 (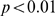), showing that the energetics of the muscle pairs are greater than the sum of the energetics attained by individual activation. This is qualitatively compatible with the model predictions plotted in Figure 4 and is in support of Hypothesis 2. This implies the possibility that energetic synergies may be achieved by a muscle-actuated system to enhance their energetic performance at particular frequency ranges.

In the experimental measurements above, the absolute power value of the muscles, normalized by muscle mass, ranged between 17 [W/kg] and 81 [W/kg] at the optimal conditions.

## Discussion

The role of active and passive muscle impedance, particularly stiffness properties, has been studied intensively in the neuromechanics and motor control literature from the perspective of stability of posture and movement. The main focus of this work is to extend this literature to include the study of muscle mechanical energetics, particularly in the context of periodic motions. We focused on the representative problem of driving a mass-spring-damper by either a single muscle or a pair of antagonist muscles. This setup can be considered as an idealization of a single degree-of-freedom joint.

### Resonance Tuning

One consequence of explicitly accounting for muscle-load interaction is the increase in the optimal stimulation frequency of the coupled system relative to the natural frequency of the uncoupled load. This is captured by Figure 3 where the maximal power was generated at a frequency higher than the uncoupled natural frequency of the load, which directly supports Hypothesis 1. This is shown computationally (Figure 3A & 3B) where it is possible to scan the range of oscillation frequencies systematically to search for the frequency of peak power generations, and also experimentally (Figures 3C & 3D) where it is possible to do so only at select frequencies chosen to show the location of peak power. The increase in optimal power generation frequency is not an unexpected result since the stiffness contributions of the muscles should couple in with the overall frequency of the load. What this enables, however, is that resonance conditions can be tuned relative to the desired frequency of oscillation via an appropriate muscle activation pattern.

Taken to the limit of zero load stiffness, we conjecture that this feature potentially enables creating resonance conditions out of non-resonant loads. The biomechanics of natural loads in many biological systems are non-resonating. Consider, as an example, the motion of a swimming fish. The external restoring force on a fish's body is negligible, therefore the sideways bending dynamics can be considered non-resonant. In the presence of muscle activation, however, significant activation modulate stiffness is added to the system, which can be tuned to the desired oscillatory frequency of the undulating motion. The importance of body bending stiffness in relation to the undulating frequency and speed of swimming fish has been reported in [32], [33].

### Antagonist Collaboration

Another consequence of the coupling between muscle impedance and load dynamics pertains to energetic synergies that are observed in systems driven by multiple muscle systems. When multiple muscles act jointly on a common load, each muscle contributes to the effective load observed by the other muscles acting on that load. This contribution can be strongly modulated by the neural input to the muscles.

Taking the simplest case of two antagonist muscles acting in parallel on a common load, Figure 5 shows that a pair of muscles can generate more power on a common load than the sum of them acting individually. The margins of collaboration were much higher than those theoretically predicted with impedance-free actuators. For a pair of identical impedance-free actuators, the ratio 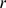 is exactly 2 at all frequencies of oscillation. When one impedance-free actuator is capable of producing more force than the other, the ratio 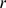 ranges between 1 and 2, but never exceeds 2. The maximal value of 2 is achieved if the two muscles provide equal forces, and the minimal value of 1 is approached as the relative contributions of the two muscles vary widely. Ratios greater than 2, as demonstrated in the 4 Hz oscillation case (shown in Figure 5C), and as demonstrated in the maximal values of Figure 4B, are in direct support of Hypothesis 2, and can only be achieved if additional muscle properties are introduced, such as activation dependent impedance.

### An Impedance Matching Interpretation

Our findings may be interpreted in the context of the engineering notion of impedance matching. In engineering systems, impedance matching plays an essential role when it is desired to maximize power transfer between two dynamical systems. When a power source is connected in series with a load (in a Thevenin equivalent connection), maximal power transfer occurs when the internal impedance of the source is equal to the complex conjugate of the load impedance [34]. In a similar manner, neural activation of muscle modulates its stiffness to allow matching of muscle mechanical impedance to that of the load. When such a condition occurs, the power transfer is maximized. This implies that the mechanical work achieved by a single muscle is highly affected by the activation pattern of antagonist muscles, because such antagonist muscles form part of the load on the agonist muscle, and therefore the energetics of muscle-actuated systems must be considered holistically.

The impedance of a linear mass-spring-damper load (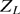) is the transfer function relating the velocity (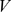) and force (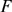) applied on the load, and can be expressed as
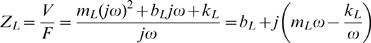
where 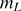, 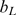, and 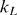 are the mass, damper and spring coefficients of the load. Assuming that the source is primarily dominated by stiffness terms, as is the case of a bilinear muscle model, the impedance of the source (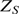) is:
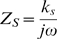
Therefore, for this source impedance, which is purely reactive, we do not have the ability to arbitrarily change the phase. To maximize the power transfer from the source to the load, impedance matching conditions require that the reactive part of the source impedance is negative the reactive part of the load impedance [34]. Therefore
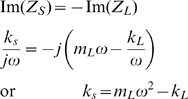
Under such conditions, the total system natural frequency becomes
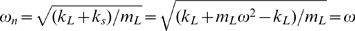
which implies that the source stiffness is chosen so that the natural frequency 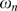 of the system matches the desired oscillation frequency 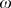.

Therefore, as the muscle pair modulates net stiffness 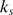, to a value that matches the desired load impedance, energetic advantages can be attained. Clearly there are limitations to the efficacy of impedance matching in helping maximize workloop energetics. For a pair of antagonist muscles to tune their stiffness to match the reactive impedance component of the load, certain amounts of co-contraction may be required. This was observed computationally with the time overlap of the control signals (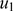 and 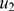). While co-contraction may attain the desired frequency tuning, it will decrease the peak-to-peak net forces produced by the muscle pair. Beyond a certain break-even point, the peak-to-peak forces will be greatly diminished to the point that impedance matching becomes non-optimal.

### Implications on Organismal Motor Control

Research in organismal motor control and biomechanics has reported extensively on the modulation of stiffness in limbs to enhance postural and dynamic stability. Our findings here provide further motivation to hypothesize that the central nervous system may utilize impedance matching as a means to enhance energetics against external loads. Prior studies support the notion that muscle stiffness is modulated to attain resonance tuning, though none have made an explicit energetic connection. Most of these investigations have focused on arm movements. In the context of rhythmic movements, perhaps the clearest evidence was provided in [35], where forearm stiffness was found to increase quadratically with oscillation frequency, and that the stiffness was minimal at the resonance of the load. It was shown that by increasing the oscillation frequency above the load resonance, the arm stiffness increased in a manner that created resonance of the arm-load system. In other studies [36]–[38], surface EMG measurements in horizontal arm reaching movements have shown that the overall co-contraction levels increase with increasing frequency of oscillation, and that co-activation increases with the square of frequency. Furthermore, in [39], neuromuscular models of the forearm that predict qualitative resonance tuning behavior in rhythmic oscillations were proposed. These arguments have also been extended to the context of of non-rhythmic movements by comparing the average forearm stiffness during reaching tasks with the fundamental frequency content of these movements [40].

The degree to which impedance matching is utilized by organisms specifically for energetic purposes remains to be addressed in future studies. Using antagonist activation of variable impedance actuators can enable the central nervous systems to learn optimal impedances that, when coupled with external loads, can provide higher energetics. Viewed from this perspective, activation dependent muscle impedance may be regarded as a favorable biomechanical property. Furthermore, this postulates that the mechanical energetics of individual muscles cannot be directly summed to estimate the total energetics of a multiple-muscle system.

## Supporting Information

Figure S1 Identification of the bilinear model for muscle contractile force. (A) To explore contractile response over a wide range of muscle velocities and positions, oscillatory motions were imposed on the muscles (shown as circles in the position-velocity space). Each circle represents a particular oscillation, with larger circles representing larger amplitudes. Electrical stimulation is triggered at the points indicated by the red asterisks. These were repeated for oscillations at various frequencies, ranging from 1–6 Hz. (B) Typical force trajectories showing modulation of contractile force (as the muscle undergoes oscillations). Experimental measurements shown in black on left, bilinear model estimates shown in blue on right. Red astersisks indicated electrical stimulation trigger points. (C) Contribution of individual model terms to the overall model fit. The bar labeled “All” shows model prediction when all terms from the generalized impedance model (Equation 13) are included. The bar labeled “Bilinear” includes only the bilinear terms (Equation 3). All terms except for the *Bx*, *C* and *Dx* can be neglected with minimal e ffects on model accuracy. Data shown are means and standard deviations from 7 muscles. (D) Left: an isometric twitch used to estimate activation states. Right: estimated activation states based on the normalized twitch profile.
(2.38 MB TIF)

Text S1 Supporting material text.
(0.19 MB PDF)

## References

[1] Nishikawa K, Biewener AA (2007). Neuromechanics: an integrative approach for understanding motor control.. Integr Comp Biol.

[2] Dickinson MH, Farley CT, Full RJ, Koehl MAR, Kram R (2000). How animals move: An integrative view.. Science.

[3] Rolf Pfeifer ML, Iida F (2007). Self-organization, embodiment, and biologically inspired robotics.. Science.

[4] Hogan N (1984). Adaptive control of mechanical impedance by coactivation of agonist muscles.. IEEE Trans Automat Contr.

[5] Hogan N (1985). The mechanics of multi-joint posture and movement control.. Biol Cybern.

[6] Full R (1998). Energy absorption during running by leg muscle in a cockroach.. J Exp Biol.

[7] Olson JM, Marsh RL (1998). Activation patterns and length changes in hindlimb muscles of the bullfrog rana catesbeiana during jumping.. J Exp Biol.

[8] Peplowski M, Marsh RL (1997). Work and power output in the hindlimb muscles of cuban tree frogs *Osteopilus septentrionalis* during jumping.. J Exp Biol.

[9] Biewener AA, Gillis GB (1999). Dynamics of muscle function during locomotion: Accomodating variable conditions.. J Exp Biol.

[10] Hogan N (1988). On the stability of manipulators performing contact tasks.. IEEE J of Robotics and Automation.

[11] Franklin DW, Milner TE (2003). Adaptive control of stiffness to stabilize hand position with large loads.. Exp Brain Res.

[12] Perreault EJ, Kirch RF, Crago PE (2002). Voluntary control of static endpoint stiffness during force regulation tasks.. J Neurophysiol.

[13] Josephson R (1985). Mechanical power output from striated muscle during cyclic contraction.. J Exp Biol.

[14] Roberts TJ (2002). The integrated function of muscles and tendons during locomotion.. Comparative Biochemistry and Physiology Part A.

[15] Tobalske BW, Hedrick TLD, P K, Biewener AA (2003). Comparative power curves in bird flight.. Nature.

[16] Josephson RK (1993). Contraction dynamics and power output of skeletal muscle.. Annu Rev Physiol.

[17] Marsh RL (1999). How muscles deal with real-world loads: The influence of length trajectory on muscle performance.. J Exp Biol.

[18] Stevens ED (1996). The pattern of stimulation influences the amount of oscillatory work done by frog muscle.. Journal of Physiology.

[19] Ahn A, Full R (2002). A motor and a brake: Two leg extensor muscles acting at the same joint manage energy differently in a running insect.. J Exp Biol.

[20] Luiker E, Stevens ED (1993). Effect of stimulus train duration and cycle frequency on the capacity to do work in the pectoral fin of muscle of the pumpkinseed sunfish, *Lopomis gibbosus*.. Can J of Zool.

[21] D Syme EDS (1989). Effect of cycle frequency and excursion amplitude on work done by rat diaphragm muscle.. Can J of Zool.

[22] Lutz G, Rome L (1994). Built for jumping: The design of the frog muscular system.. Science.

[23] J Renaud EDS (1983). A comparison between field habits and contractile performance of frog and toad sartorius muscle.. J Comp Physiol.

[24] Roberts TJ, Scales JA (2002). Mechanical power output during running accelerations in wild turkeys.. Comparative Biochemistry and Physiology Part A.

[25] Farahat W, Herr H (2005). An apparatus for characterization and control of isolated muscle.. IEEE Trans Neural Syst Rehab Eng.

[26] Hogan N (1990).

[27] Crago PE (1992). Muscle input-output model: The static dependence of force on length, recruitment and firing period.. IEEE Trans Biomed Eng.

[28] Farahat WA (2007). Optimal workloop Energetics of Muscle-Actuated Systems..

[29] Bryson AE, Ho YC (1975). Applied Optimal Control: Optimization, Estimation and Control.

[30] Smith DR (1974). Variational Methods in Optimization.

[31] Stevens ED (1988). Effect of pH and stimulus phase on work done by isolated frog sartorius muscle during cyclical contraction.. J Muscle Research and Cell Motility.

[32] Matthew J, McHenry CAP, John H, Long J (1995). Mechanical control of swimming speed: Stiffness and axial wave form in undulating fish models.. J Exp Biol.

[33] John H Long J, Nipper KS (1996). The importance of body stiffness in undulatory propulsion.. Amer Zool.

[34] Balabanian N, Bickart TE (1997).

[35] Abe MO, Yamada N (2003). Modulation of elbow joint stiffness in a vertical plane during cyclic movement at lower or higher frequencies than natural frequency.. Exp Brain Res.

[36] Pailhous J, Bonnard M, Coyle T (1996). Autonomy versus forcing in the organization of human rhythmic forearm movements.. C R Acad Sci III.

[37] Feldman AG (1980). Superposition of motor programs i. rhythmic forearm movements in man.. Neuroscience.

[38] Latash ML (1992). Virtual trajectories, joint stiffness, and changes in the limb natural frequency during single-joing oscillatory movements.. Neuroscience.

[39] BW Verdaasdonk, HFJM Koopman, FCT VanderHel (2007). Resonance tuning in a neuro-musculo-skeletal model of the forearm.. Biological Cybernetics.

[40] Bennet DJ (1993). Torques generated at the human elbow joint in response to constant position errors imposed during voluntary movements.. Exp Brain Res.

