# Supplementary figures and images for: Optimal Workloop Energetics of Muscle-Actuated Systems: An Impedance Matching View
Source: PLoS Comput Biol. 2010 Jun 3;6(6):e1000795. doi: 10.1371/journal.pcbi.1000795 (PMC2880559; doi:10.1371/journal.pcbi.1000795)

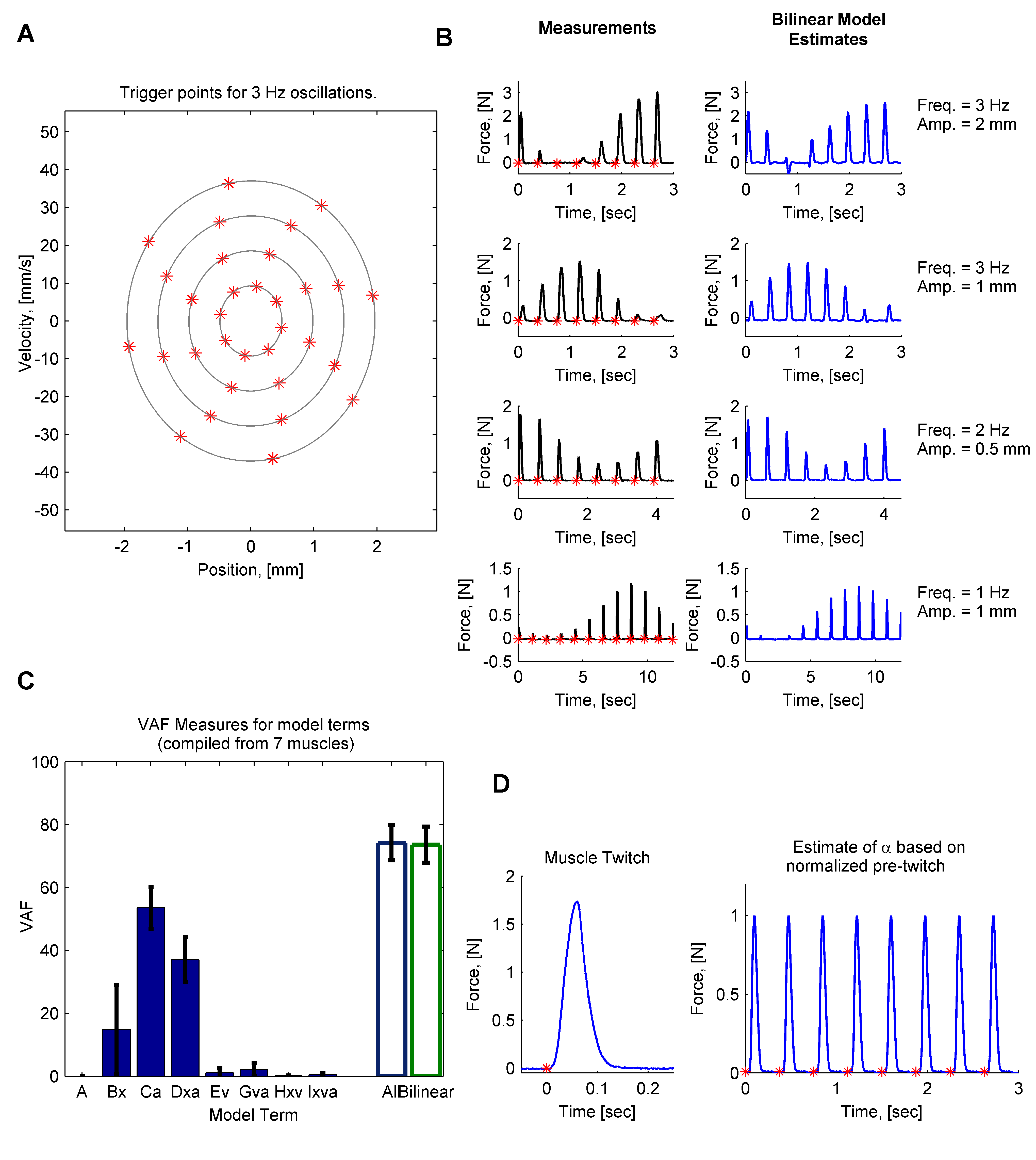

Supplement: Figure S1 — Identification of the bilinear model for muscle contractile force. (A) To explore contractile response over a wide range of muscle velocities and positions, oscillatory motions were imposed on the muscles (shown as circles in the position-velocity space). Each circle represents a particular oscillation, with larger circles representing larger amplitudes. Electrical stimulation is triggered at the points indicated by the red asterisks. These were repeated for oscillations at various frequencies, ranging from 1–6 Hz. (B) Typical force trajectories showing modulation of contractile force (as the muscle undergoes oscillations). Experimental measurements shown in black on left, bilinear model estimates shown in blue on right. Red astersisks indicated electrical stimulation trigger points. (C) Contribution of individual model terms to the overall model fit. The bar labeled “All” shows model prediction when all terms from the generalized impedance model (Equation 13) are included. The bar labeled “Bilinear” includes only the bilinear terms (Equation 3). All terms except for the Bx, C and Dx can be neglected with minimal e ffects on model accuracy. Data shown are means and standard deviations from 7 muscles. (D) Left: an isometric twitch used to estimate activation states. Right: estimated activation states based on the normalized twitch profile. (2.38 MB TIF) [file pcbi.1000795.s001.tif]
