# Supplementary material for: Optimal Workloop Energetics of Muscle-Actuated Systems: An Impedance Matching View
Source: PLoS Comput Biol. 2010 Jun 3;6(6):e1000795. doi: 10.1371/journal.pcbi.1000795 (PMC2880559; doi:10.1371/journal.pcbi.1000795)
Supplement: Text S1 — Supporting material text. (0.19 MB PDF) [file pcbi.1000795.s002.pdf]

Supporting Material

Bilinear Model for Muscle Force

In the Materials and Methods section, we assumed that for the oscillatory motions considered, muscle mechanical impedance can be approximated by a bilinear model. The bilinear model described by Equation (3), is repeated here:

$$F_i \quad = \quad A_i + B_i x_i + C_i \alpha_i + D_i x_i \alpha_i \qquad\qquad i \in \{1, 2\}$$

How did we arrive at this approximation, and what is the accuracy of this model? Here we provide a brief summary based on [1].

To arrive at a muscle impedance model, we conducted measurements to characterize the mechanical impedance of the *Plantarus longus* muscles, similar to the ones used in our investigation (but not the particular muscles used to test the hypothesis because of the limited fatigue life of the muscles). We used the same materials and methods described in the main text. This includes muscle type, harvesting procedure, and the use of the servo-mechanical muscle testing apparatus. There were two differences, however, due to the fact that measurements focused on uncovering the dependence of muscle force on mechanical state, rather than estimating workloop energetics. First, sinusoidal motions were imposed on the muscles, and electrical stimulation was delivered at predetermined points in time. Thus, the experimental setup was akin to Figure 1A in the main text. The electrical stimulus points were not synchronized with the oscillations, but rather were chosen to span various displacement-velocity combinations along the sinusoidal trajectory of the muscle. Thus, the timing of electrical stimulation trigger points was uncorrelated with the imposed motion. Sample oscillatory motions are illustrated in Figure S1A. The second difference is that the electrical stimulus parameters were fixed, and were not dependent on prior optimal control computations. The electrical stimulus parameters were set to at duration of 50 msec, consisting of 10 spikes at 200 Hz, with a spike duration of 100 micro-seconds.

To explore the relative importance of various impedance factors, we used the acquired data to fit a general impedance function in the form of:

$$F = A + Bx + C\alpha + Dx\alpha + Ev + Gv\alpha + Hxv + Ixv\alpha \qquad\qquad (S1)$$

While additional higher-order model terms could have been added, this general model can capture many facets of the impedance characteristics of the muscle, including passive stiffness (Bx), activation dependent stiffness ($Dx\alpha$), passive damping (Ev), activation dependent damping ($Gv\alpha$), visco-elasticity (Hxv) and activation dependent visco-elasticity ($Ixv\alpha$).

This generalized model was fit to data from 7 different muscles, with amplitudes of motion ranging between 0.5 and 2.0 mm, and frequencies of oscillation ranging between 1 and 7 Hz. We found that this generalized model (Equation (S1)) captured $74.2\% \pm 5.5\%$ of the temporal variance in the force generated.

To further simplify this model, and to assess the importance of each of those relative terms, we performed linear regression to fit the relationship between the observed force and the individual regressors (x, $\dot{x}$ and α), and found that the dominant terms characterizing muscle response were the bilinear terms, namely Bx, $C\alpha$ and $Dx\alpha$. Figure S1C shows the contributions of each of the individual regressors to the variance of the output force. The results show that the bilinear approximation $F \approx A + Bx + C\alpha + Dx\alpha$ captured $73.6\% \pm 5.7\%$ of the total variance in muscle force, which is a minor degradation in accuracy compared to the model including the damping and visco-elastic terms. The parameters were normalized by the value of the C coefficient to account for the differences in muscle size. The normalized parameters used in the optimization were: $A = 0$, $B = 0.016$, $C = 1$, and $D = 0.727$.

Fitting the parameters required knowledge of the activation α. While estimates of α from Equations (1) and (2) are possible, errors in the activation dynamics model may contribute to errors in the experimental identification of the impedance model. In order to minimize this effect, for each muscle we

used a normalized profile of its isometric contraction as a representation of the activation dynamics. In doing so, we made the assumption that the activation dynamics did not vary with the length or velocity of the muscle. However, the normalized isometric force profile was used to determine the parameters of Equations (1) and (2). The parameters of the activation model used were $p_{\alpha_i} = -62$, $p_{\beta_i} = -62$ and $p_{u_i} = 3947$, which results in a step input gain of unity, and a twitch rise and fall time of 125 msec.

We do not necessarily advocate that this model be used as a general muscle model since it has been tested only in the context of oscillatory motions with bursting contractions. However, in those particular conditions, it provided a reasonably descriptive approximation to the muscle force that is also mathematically tractable enough to be used for the optimal control formulation. What is evident from the model, however, is that the dependence of muscle force on velocity was found to be minimal. Whether this is a particular feature of that muscle, or whether it is a resultant effect of this particular experimental paradigm can be a topic of further research.

Computation of Optimal Control

The solution of the 2-point boundary value problem (Equation (7)) is non-trivial, primarily due to the discontinuous nature of the control. Even with the addition of the quadratic term, the hard bounds on the control lead to the failure of many of the standard solution algorithms. We solve this issue via the use of penalty methods. Penalty methods simplify the solution of a constrained optimization problem by converting it to a sequence of unconstrained optimization problems [2]. This sequence of problems is solved, with an increasing penalty function added for violating the constraints. In the limit, as the value of the penalty weight increases, the solutions will converge to the optimal solution of the constrained problem.

We applied penalty methods to the optimal control problem in the following manner. We would like to make sure that the control is limited to the admissible bounds, i.e. $u_i \leq u_{max}$ and $u_i \geq u_{min}$ for all t. Therefore the penalties are imposed if the control u_i violates those bounds. We also use quadratic penalties in the form of

$$p(u_i) = \begin{cases} (u_i - u_{max})^2 & \text{if } u_i > u_{max} \\ 0 & \text{if } u_i \text{ is feasible} \\ (u_{min} - u_i)^2 & \text{if } u_i < u_{min} \end{cases}$$

Therefore, the optimal control problem takes the following form:

$$\begin{aligned} J^* &= \min_{\mathbf{u}} \int_0^T -F_{net}\dot{x} + c_k p(u - u_{max}) + c_k p(u_{min} - u) dt \\ \text{s.t.} \quad & \dot{\mathbf{x}} = \mathbf{f}(\mathbf{x}, \mathbf{u}) \\ & \mathbf{x}(0) = \mathbf{x}(T) \\ \text{where} \quad & \eta \geq 0 \\ & c_k \geq 0 \\ & p(y) = \begin{cases} 0 & \text{for } y < 0 \\ y^2 & \text{for } y \geq 0 \end{cases} \end{aligned}$$

Recall that the optimal control was a bang-bang control strategy, implying that depending on the sign of the switching function, it is optimal to push the control signal as far out as possible. With the penalty methods, a softened bound becomes optimal to push the control signal as far out until the penalty dominates.

Consider the case if the resulting optimal control is between u_{min} and u_{max}. Then $p(u_i) = 0$. Therefore,

$$u_1^* = \arg\min_{u_1} \left\{\lambda_2 p_{u_1} + \eta \mathbf{u}^T \mathbf{u}\right\}$$
$$\Rightarrow u_1^* = \frac{-\lambda_2 p_{u_1}}{2\eta}$$

$$u_2^* = \arg\min_{u_2} \left\{\lambda_4 p_{u_2} + \eta \mathbf{u}^T \mathbf{u}\right\}$$
$$\Rightarrow u_2^* = \frac{-\lambda_4 p_{u_2}}{2\eta}$$

If $\frac{-\lambda_2 p_{u_1}}{2\eta} > u_{max}$ or $\frac{-\lambda_4 p_{u_2}}{2\eta} > u_{max}$, then $p(u_i) = (u_i - u_{max})^2$. Therefore,

$$u_1^* = \arg\min_{u_1} \left\{\lambda_2 p_{u_1} + \eta \mathbf{u}^T \mathbf{u} + c_k(u_1 - u_{max})\right\}$$
$$\Rightarrow u_1^* = \frac{2c_k u_{max} - \lambda_2 p_{u_1}}{2(\eta + c_k)}$$

$$u_2^* = \arg\min_{u_2} \left\{\lambda_4 p_{u_2} + \eta \mathbf{u}^T \mathbf{u} + c_k(u_2 - u_{max})\right\}$$
$$\Rightarrow u_2^* = \frac{2c_k u_{max} - \lambda_4 p_{u_2}}{2(\eta + c_k)}$$

If $\frac{-\lambda_2 p_{u_1}}{2\eta} < u_{min}$ or $\frac{-\lambda_4 p_{u_2}}{2\eta} < u_{min}$, then $p(u_i) = (u_{min} - u_i)^2$. Therefore,

$$u_1^* = \arg\min_{u_1} \left\{\lambda_2 p_{u_1} + \mathbf{u}^T \mathbf{u} \alpha + c_k(u_{min} - u_1)^2\right\}$$
$$\Rightarrow u_1^* = \frac{2c_k u_{min} - \lambda_2 p_{u_1}}{2(\eta + c_k)}$$

$$u_2^* = \arg\min_{u_2} \left\{\lambda_4 p_{u_2} + \mathbf{u}^T \mathbf{u} \alpha + c_k(u_{min} - u_2)^2\right\}$$
$$\Rightarrow u_2^* = \frac{2c_k u_{min} - \lambda_4 p_{u_2}}{2(\eta + c_k)}$$

In summary:

$$u_1^* = \begin{cases} \dfrac{-\lambda_2 p_{u_1}}{2\eta} & \text{if } u_{min} < \dfrac{-\lambda_2 p_{u_1}}{2\eta} < u_{max} \\[2ex] \dfrac{2c_k u_{max} - \lambda_2 p_{u_1}}{2(\eta + c_k)} & \text{if } \dfrac{-\lambda_2 p_{u_1}}{2\eta} > u_{max} \\[2ex] \dfrac{2c_k u_{min} - \lambda_2 p_{u_1}}{2(\eta + c_k)} & \text{if } \dfrac{-\lambda_2 p_{u_1}}{2\eta} < u_{min} \end{cases}$$

$$u_2^* = \begin{cases} \dfrac{-\lambda_4 p_{u_2}}{2\eta} & \text{if } u_{min} < \dfrac{-\lambda_4 p_{u_2}}{2\eta} < u_{max} \\[2ex] \dfrac{2c_k u_{max} - \lambda_4 p_{u_2}}{2(\eta + c_k)} & \text{if } \dfrac{-\lambda_4 p_{u_2}}{2\eta} > u_{max} \\[2ex] \dfrac{2c_k u_{min} - \lambda_4 p_{u_2}}{2(\eta + c_k)} & \text{if } \dfrac{-\lambda_4 p_{u_2}}{2\eta} < u_{min} \end{cases}$$

We solve this softened version of the problem numerically. We start with low values for c_k, and increase it until $u_i(t)$ violates the bounds constraints to within acceptable errors.

Solving the 2-point boundary value problem The 2-point boundary value problem is solved using the MATLAB solver `bvp4c` for increasing values of c_k. This solver descretizes the solution space to via an adaptive mesh, and fits piecewise cubic functions to each segment. The residuals in the interface points have to match the differential equation, and the boundary conditions. A description of the algorithm is found in [3].

References

1. Farahat WA (2007) Optimal workloop Energetics of Muscle-Actuated Systems. Ph.D. thesis, Massachusetts Institute of Technology (MIT).

2. Fruend R (2004). Lecture notes on nonlinear programming (course notes for 6.252). Massachusetts Institute of Technology.

3. Shampine LF, Kierzenka J (2000). Solving boundary value problems for ordinary differential equations in MATLAB using `bvp4c`.
